# Ditelluride-Bridged PEG-PCL Copolymer as Folic Acid-Targeted and Redox-Responsive Nanoparticles for Enhanced Cancer Therapy

**DOI:** 10.3389/fchem.2020.00156

**Published:** 2020-02-28

**Authors:** Zekun Pang, Jiayan Zhou, Chunyang Sun

**Affiliations:** Department of Radiology and Tianjin Key Laboratory of Functional Imaging, Tianjin Medical University General Hospital, Tianjin, China

**Keywords:** ditelluride linkage, redox responsive, targeted nanoparticle, drug delivery, cancer therapy

## Abstract

The development of the nanosized delivery systems with targeting navigation and efficient cargo release for cancer therapy has attracted great attention in recent years. Herein, a folic acid (FA) modified PEGylated polycaprolactone containing ditelluride linkage was synthesized through a facile coupling reaction. The hydrophobic doxorubicin (DOX) can be encapsulated into the polymeric micelles, and such nanoparticles (F-TeNP_DOX_) exhibited redox-responsive drug release under abundant glutathione (GSH) condition due to the degradation of ditelluride bonds. In addition, flow cytometric analyses showed that the FA ligands on F-TeNP_DOX_ could facilitate their cellular uptake in 4T1 breast cancer cells. Therefore, F-TeNP_DOX_ led to the promoted drug accumulation and enhanced growth inhibition on 4T1 tumor *in vivo*. The obtained results suggest F-TeNP_DOX_ excellent potential as nanocarriers for anticancer drug delivery.

## Introduction

Doxorubicin (DOX) is one of the widely used chemotherapeutic agents to treat different tumors (Rivankar, 2014). However, its clinical applications are hampered by the lack of selectivity, severe toxicity, and reduced anticancer efficacies (Olson and Mushlin, 1990; Kang et al., 1996). Over the past decades, it is found that nanoscale drug delivery vehicles can help conventional chemotherapeutic drugs to overcome this limitation (Blanco et al., 2015; Xiong et al., 2016). The nanoparticles are able to increase drug accumulation in tumors via enhanced permeability and retention (EPR) effect (Maeda et al., 2013; Wilhelm et al., 2016). Among the nanoparticles reported, most of them have been decorated by PEGylation to improve the biocompatibility and stability after the administration (Butcher et al., 2016; Huckaby and Lai, 2018; Suh et al., 2019). Although outside PEG layer can validly preserve nanoparticles from rapid clearance by the reticuloendothelial system (RES) to extend their blood circulation, its application would significantly impede cellular uptake by tumor cells and result in unsatisfactory therapeutic efficiency (Zhu et al., 2012; Fang et al., 2017; Chen et al., 2018). Accordingly, optimization of the carriers with active targeting moieties are of great interest in drug delivery field. The targeting ligands can recognize the over-expressed receptors on cancer cells, thus facilitate cell internalization via receptor-ligand mediated endocytosis (Lazarovits et al., 2015; Kosmides et al., 2017). For example, high-affinity folate receptor is a glycosylphosphatidylinositol-linked cell surface receptor and usually expressed at elevated levels on tumoral cells. Accordingly, folic acid, which is a cheap, chemically stable and water-soluble B vitamin and could specifically recognize folate receptor, has become a commonly used ligand for convenient tumor navigation. Up to now, a series of FA modified delivery system have been developed for active targeting to cancer cells (Gabizon et al., 2004; Fathi et al., 2017; Liu et al., 2019). Following the FA decoration and attaching to the receptors, the nanocarriers would be internalized into cancer cells through the endocytotic pathway to increase intracellular drug content (Zwicke et al., 2012; Cheng et al., 2017). Additionally, other functional targeting ligands, such as arginine-glycine-aspartic acid sequences (RGD), hyaluronic acid, prostate-specific membrane antigen sequences (PSMA) have also been proven to promote drug accumulation in different types of tumor after appropriate modification on nanoparticles (Chai et al., 2019; Ma et al., 2019; Yoo et al., 2019; Zhou et al., 2019).

To amplify the therapeutic benefits, the ideal nanocarriers should boost the drug release rapidly after entering the cells (Deepagan et al., 2018; Wang et al., 2018; Sangtani et al., 2019). Unfortunately, it is difficult for conventional nanocarriers to achieve timely on-demand drug release, leading to marginal drug exposure to cancer cells. The stimuli-responsive drug release systems, which would be degraded by responding with the exogenous physicochemical stimulus (pH, reducing agent, light, temperature, etc.) in tumor microenvironment, garnered considerable attention during the last decade (El-Sawy et al., 2018; Jiménez-Balsa et al., 2018; Li et al., 2018; Qu et al., 2018). Among numerous design, disulfide linkages were widely utilized to fabricate responsive systems due to the dramatical difference of the reducing glutathione (GSH) concentration between extracellular and intracellular microenvironment (ca. 2 μM vs. ca. 10 mM) (Wu et al., 2015; Lee et al., 2016; Zhang et al., 2016). In recent years, an increasing number of studies have given importance to tellurium-containing nanoparticles. As one of the chalcogens, the chemical property of tellurium is likely to be similar to sulfur and selenium. However, because of its weaker electronegativity and stronger radius, tellurium has lower bond energy. In contrast to S–S bond (240 kJ/mol) and Se–Se bond (192 kJ/mol), the energy of Te–Te bond is only 149 kJ/mol, which reveals that the ditelluride-containing nanoparticles are more prone to be reacted by reducing stimulus (Chivers and Laitinen, 2015; Wang et al., 2016). For instance, Xu et al. proposed and synthesized a series of telluride-containing polymer which is sensitive to higher ROS concentration or gamma radiation (Cao et al., 2014, 2015a,b; Wang et al., 2015). Furthermore, Zhang's group made an effort to design another ditelluride-containing poly(ether-urethane) drug delivery system as well, suggesting the excellent potential for antitumor activity (Fang et al., 2015). Therefore, it might be a promising strategy to develop a targeted ditelluride-containing nanocarriers to achieve cascade cellular uptake and intracellular drug release for efficient cancer treatment.

**Figure 1:**
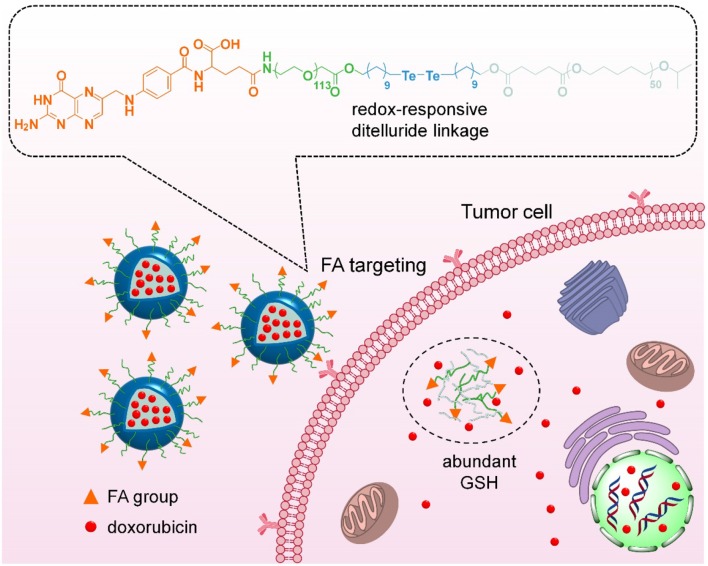
Scheme of targeting drug delivery and GSH-responsive drug release for efficient cancer therapy.

Herein, we have developed an integrated nanoparticles platform containing FA ligands and ditelluride bonds for active tumor targeting and GSH-responsive drug release (Figure 1). It can self-assembly in aqueous solution to encapsulate the DOX, and the hydrophilic PEG shell can prolong DOX circulation in blood. Following the passive extravasation to tumor tissues via EPR effect, FA ligands bared on the surface of nanoparticles can facilitate the endocytosis for FA receptor specific expression tumor cells. Subsequently, F-TeNP_DOX_ would be swelled rapidly in a reducing environment after the cleavage of bridged bonds to trigger drugs release. As a result, the enhanced inhibition on tumor growth can be achieved.

## Materials and Methods

### Materials

Epsilon-caprolactone (ε-CL, 99%) was purchased from Alfa Aesar Chemical Company, Inc., and used after CaH_2_ treatment and distillation. Carboxyl group modified poly(ε-caprolactone) (PCL-COOH) with 5700 average molecular weights was synthesized using aluminum isopropoxide as the initiator according to reported method (Dzienia et al., 2017). Poly(ethylene glycol) monomethylether (PEG, Mw = 5000) and 11-bromoundecanol were obtained from Sigma-Aldrich. The block copolymers of poly(ethylene glycol) monomethylether and poly(ε-caprolactone) (PEG-*b*-PCL) was synthesized as previously reported and the average molecular weights of PCL was 5930 (Han et al., 2015). FA-PEG-*b*-PCL (PEG Mw = 5000, PCL Mw = 5240) were obtained from Xi'an Ruixi Biological Technology Co., Ltd. Disodium telluride and di(1-hydroxylundecyl) ditelluride were prepared according to the previous reports (Wang et al., 2016). 3-(4,5-dimethylthiazol-2-yl)-2,5-diphenyl tetrazolium bromide (MTT) was purchased from Energy Chemical Co., Ltd. (Shanghai, China). Doxorubicin hydrochloride (DOX·HCl) was obtained from Beijing HVSF United Chemical Materials Co., Ltd. All other reagents were purchased from Shanghai Aladdin Bio-Chem Technology Co., LTD. and used as received.

### Synthesis of Ditelluride-Bridged Block Copolymer With FA Ligand

The targeted diblock copolymer containing ditelluride linker (FA-PEG-*TeTe*-PCL) was synthesized via coupling reaction among FA-PEG-COOH, di(1-hydroxylundecyl) ditelluride and PCL-COOH. Initially, FA-PEG-COOH (0.37 mmol, 2.03 g) and di(1-hydroxylundecyl) ditelluride (1.1 mmol, 0.66 g) were mixed in 20 mL of anhydrous CH_2_Cl_2_ in a three-necked flask at 25°C, then DCC (0.56 mmmol, 115.5 mg) and DMAP (0.56 mmol, 62.8 mg) was added drop wise under nitrogen atmosphere. After reacting at rt for 24 h, the byproduct was removed by filtration. The filtrate was concentrated and precipitated into cold diethyl ether three times to obtain the FA-PEG-*TeTe*-OH. Next, the FA-PEG-*TeTe*-OH (0.19 mmol, 1.17 g), PCL-COOH (0.09 mmol, 0.4 g), DCC (0.14 mmol, 28.9 mg), and DMAP (0.14 mmol, 15.7 mg) were dissolved in 15.0 mL of anhydrous CH_2_Cl_2_ and reacted at 25°C under N_2_. After 24 h, the mixture was filtered, concentrated and precipitated into cold diethyl ether/methanol (10:1 v/v) three times.

### Preparation of Drug-Loaded Nanoparticles

Briefly, 20.0 mg of PEG-*b*-PCL, FA-PEG-*b*-PCL, and FA-PEG-*TeTe*-PCL and 2.0 mg of DOX were mixed in 1.0 mL of DMSO and stirred for 30 min. Then 20.0 mL of ddH_2_O was dropwise added under vigorous stirring and kept for another 2 h. The organic phase was removed by dialysis against ultrapure water in dialysis tubing (MWCO = 14000). The free DOX was further removed by centrifugation at 3,000 rpm for 5 min. The obtained nanoparticles were denoted by NP_DOX_, F-NP_DOX_ and F-TeNP_DOX_, respectively. The DOX loading content was determined by UV-vis spectrophotometer at 480 nm.

### Drug Release *in vitro*

Two milliliters of NP_DOX_, F-NP_DOX_, and F-TeNP_DOX_ (with equivalent DOX concentration at 100 μg/mL) were added into dialysis tubing (MWCO 3,500 Da). Then the tubing was immersed in the 10 mL of phosphate buffer (PB, 20 mM, pH 7.4) with or without 10 mM GSH in a shaker (120 rpm/min) at 37°C. At different time points, the external PB buffer was collected, and the tubing was immersed in fresh buffer at 37°C for further detection. The DOX content in the collected sample was measured by HPLC analysis (Ma et al., 2016).

### Cellular Uptake of F-TeNP_DOX_ *in vitro*

For the FACS analysis, 4T1 or NIH-3T3 cells were seeded in 12-well plates at a density of 100,000 cells per well. After culturing for 12 h, the original medium was replaced with that containing free DOX (4 μg/mL), NP_DOX_, F-NP_DOX_ and F-TeNP_DOX_ (equivalent [DOX] = 4 μg/mL). The cells were incubated for another 4 h, and then treated with cold phosphate buffered saline (PBS, 10 mM, pH 7.4) twice and trypsin (0.25%, Gibco, Canada). The cells were harvested and resuspended in paraformaldehyde (4%, 200 μL) for flow cytometry on BD FACS Verse.

In addition, cells treated with various formulations were lysed by 1% Triton X-100 (in 250 μL of PBS) after washing with cold PBS twice. The DOX content in the lysates was measured by HPLC, and the intracellular protein content was determined by a BCA Protein Assay Kit (Pierce, Rockford, IL).

To observe the subdistribution, the 4T1 cells were seeded on coverslips in 12-well plates. The cells were incubated with medium containing free DOX (4 μg/mL), NP_DOX_, F-NP_DOX_, and F-TeNP_DOX_ (equivalent [DOX] = 4 μg/mL) for 4 h. Then, the cell nuclei was counterstained by 4′,6-diamidino-2-phenylindole (DAPI, Beyotime, China) for the cell nuclei and F-actin was counterstained by Alexa Fluor® 488 Phalloidin (Invitrogen, Carlsbad, USA), respectively. The cells were visualized using a confocal laser scanning microscope (CLSM, LMS810, Zeiss).

### Cytotoxicity of F-TeNP_DOX_ *in vitro*

To determine the cytotoxicity of NP_DOX_, F-NP_DOX_ and F-TeNP_DOX_, a MTT assay was used against 4T1 or NIH-3T3 cells. The cells were seeded in 96-well plates at 10,000 cells per well, and treated with medium containing free DOX, NP_DOX_, F-NP_DOX_, or F-TeNP_DOX_ at different concentrations. After the incubation of 12 h, the cells were further incubated with fresh DMEM medium for another incubation of 60 h. Subsequently, DMEM medium and the MTT stock solution were added to each well. The final MTT concentration is 1 mg/mL. Following further incubation for 2 h, extraction buffer (20% sodium dodecyl sulfate in 50% DMF, pH 4.7) was added and the plate was incubated at 37°C for 4 h. The mixture absorbance was measured by a Bio-Rad 680 microplate reader at 570 nm and the cell viability was calculated according to the previously reported method. The cytotoxicity of nanoparticles without DOX loading was measured similarly.

To determine the cell apoptosis, 4T1 cells were seeded into 24-well plates and incubated for 12 h. Subsequently, the cells were treated with various DOX-loaded nanoparticles as same as the MTT assay. Thereafter, the cells were treated by Annexin V-FITC apoptosis detection kit I (BD Biosciences). The results were measured using BD Accuri® C6 flow cytometer.

### Pharmacokinetics and Biodistribution of F-TeNP_DOX_

Female ICR mice were divided into 4 groups (*n* = 4), and were received *i.v*. injection with DOX, NP_DOX_, F-NP_DOX_, or F-TeNP_DOX_ ([DOX] = 10 mg/kg). After the predetermined time intervals, blood samples were collected from the retroorbital plexus of the mouse eye and the DOX content in plasma was measured via previously reported method.

To determine the DOX internalization in tumoral cells, mice bearing 4T1/GFP xenografts were administrated with DOX, NP_DOX_, F-NP_DOX_ or F-TeNP_DOX_. At 4, 12, or 24 h, the tumor tissues were harvested and digested into single cell suspension. The quantitative distribution of DOX in GFP^+^ cells were analyzed by both FACS and HPLC.

### Anticancer Efficiency of F-TeNP_DOX_ *in vivo*

The mice bearing 4T1 xenograft were randomly divided into five groups (*n* = 5). When the tumor volume was about 50 mm^3^, the mice received systemic injection every week with PBS, DOX (5.0 mg/kg), NP_DOX_, F-NP_DOX_, or F-TeNP_DOX_ ([DOX] = 5.0 mg/kg). Tumor size and body weight were monitored every 3 days. For *in vivo* biosafety assay, mice were treated daily with various formulation for 3 days ([DOX] = 5.0 mg/kg). Serum was collected for hematologic evaluation and enzyme-linked immunosorbent assay (ELISA) for alanine aminotransferase (ALT), aspartate transaminase (AST), and blood urea nitrogen (BUN).

## Results

### Synthesis and Characterization of FA-PEG-TeTe-PCL

**Figure 2:**
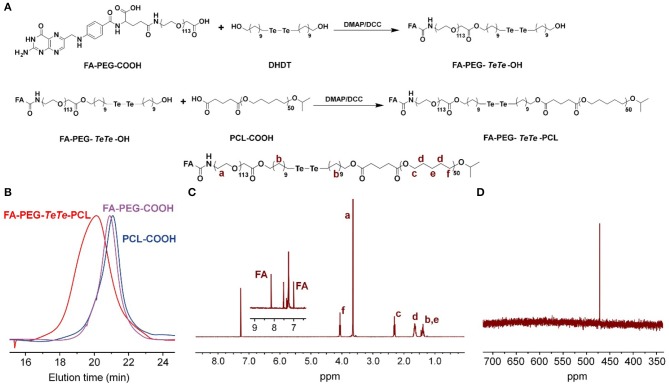
**(A)** Synthetic route of FA-PEG-*TeTe*-PCL. GPC spectrum **(B)** ^1^H NMR spectrum **(C)** and ^125^Te NMR spectrum **(D)** of FA-PEG-*TeTe*-PCL.

The synthetic route of redox-responsive ditelluride-containing copolymer was shown in Figure 2A. The FA-PEG-*TeTe*-PCL was synthesized by a two-step coupling reaction. The FA-PEG-COOH was first reacted with di(1-hydroxylundecyl) ditelluride (DHDT) to obtain FA-PEG-*TeTe*-OH. Then, FA-PEG-*TeTe*-OH was coupled with an carboxyl-modified poly(ε-caprolactone) (PCL-COOH), and the excess FA-PEG-TeTe-OH was removed by precipitating the product using methanol. The unimodal gel permeation chromatography (GPC) profile (Figure 2B) indicated an obvious shift toward higher molecular weight, suggesting successful coupling. The ^1^H NMR spectrum in Figure 2C showed the molar ratio of PEG and PCL was 1:1.07, which is close to that of the desired product. Meanwhile, ^125^Te NMR analysis in Figure 2D also presented a characteristic peak at 471.3 ppm, further demonstrating its chemical structure. In addition, the non-responsive FA-PEG-*b*-PCL and PEG-b-PCL were used as the control in the following experiments.

### Preparation and Characterization of Redox-Sensitive Nanoparticles Containing Ditelluride Linkage

**Figure 3:**
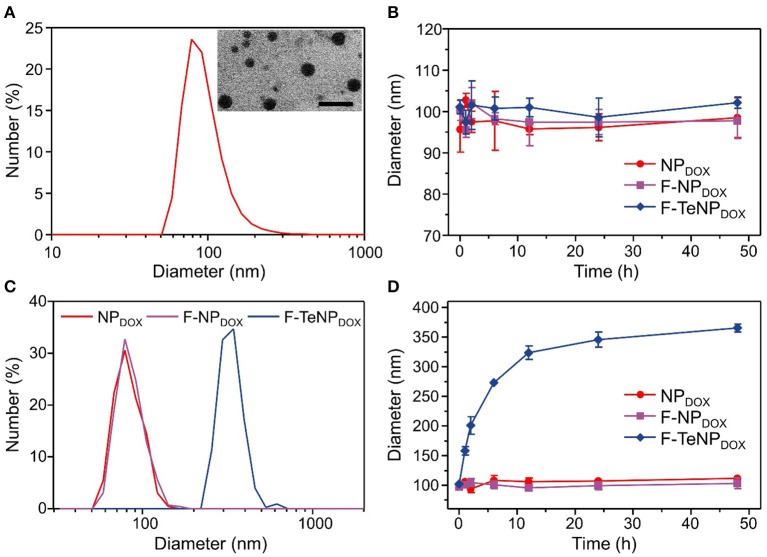
Characterization of the redox-responsive properties of DOX-loaded micelles. **(A)** Size distribution and morphology of F-TeNP_DOX_. The scale bar is 200 nm. **(B)** Change of hydrodynamic size in PBS solution (pH 7.4). **(C,D)** Diameter change of NP_DOX_, F-NP_DOX_, and F-TeNP_DOX_ with 10 mM GSH.

The FA-PEG-*TeTe*-PCL, FA-PEG-*b*-PCL and PEG-*b*-PCL could be self-assembled into micellar nanoparticles in aqueous solution, and then efficiently encapsulated hydrophobic DOX in the hydrophobic core. The fabricated micelles were denoted by F-TeNP_DOX_, F-NP_DOX_, and NP_DOX_, respectively. As shown in Figure 3A, the average hydrodynamic diameter of F-TeNP_DOX_ was found as 96.5 nm by dynamic light scattering (DLS) and polydispersity index was 0.158, whereas the nanoparticles in this size range were believed to be passively accumulated in tumors through the EPR effect (Li et al., 2010; Maruyama, 2011). Owing to the presence of hydrophilic PEG component, the size observed by transmission electron microscopy was slightly smaller than that from DLS measurement. Meanwhile, F-TeNP_DOX_ with the PEG chains on the surface showed a zeta potential of −10.7 mV. The DOX loading content and encapsulation efficiency of F-TeNP_DOX_ was determined by UV-vis spectra as 5.74 and 60.9%, respectively. In addition, both F-NP_DOX_ and NP_DOX_ exhibited comparable properties after DOX loading (Figure S1 and Table S1). Due to the smaller size and the protection from the outer PEG shell, all of three nanoparticles maintained their original diameter after incubating in PBS solution for 48 h (Figure 3B).

According to our design, the bridged ditelluride bonds in FA-PEG-*TeTe*-PCL would be selectively cleaved in a reducing environment after cellular uptake. Considering the degradation of PEG corona lead to the nanoparticles swelling (Chen et al., 2016), we measured the size change of three nanoparticles in the presence of GSH. As shown in Figure 3C, the size of the F-TeNP_DOX_ at 24 h was significantly raised to ~320 nm, while the control F-NP_DOX_ and NP_DOX_ showed no remarkable size change. Furthermore, the size change of nanoparticles under abundant GSH condition was monitored during 48 h at different time intervals. As illustrated in Figure 3D, the aggregations of F-TeNP_DOX_ was observed and the size gradually increased to approximately 365 nm. On the other hand, both control groups showed negligible size variation, in agreement with the above mentioned result. These data indicated that the ditelluride bond in F-TeNP_DOX_ could be cleaved by endogenous GSH stimulus, resulting in the detachment of the PEG layer and dissociation of the F-TeNP_DOX_ nanoparticle.

### DOX Release *in vitro*

**Figure 4:**
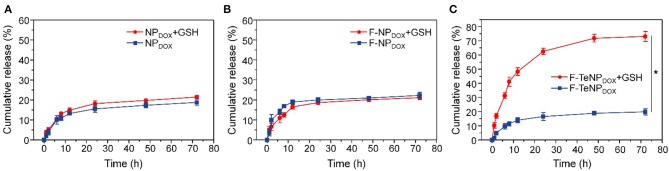
The DOX release profile from NP_DOX_ **(A)**, F-NP_DOX_ **(B)**, or F-TeNP_DOX_ **(C)** at different conditions ([GSH] = 10 mM). *p < 0.05.

It is well-verified that the degradation of the PEG shell would facilitate cargo release. Therefore, the DOX would be rapidly released from F-TeNP_DOX_ under redox conditions. To verify this hypothesis, quantitative drug release *in vitro* was investigated at 37°C in phosphate buffer (PB, 20 mM, pH 7.4) with or without 10 mM GSH, and the cumulative DOX release was monitored using HPLC. As shown in Figure 4A, the drug release rate for F-TeNP_DOX_ was significantly accelerated when treated with 10 mM GSH. There was almost 76.71 ± 2.8% of DOX and was detected after 24 h, and the continual release can be observed until the end of the experiment. On the contrary, the cumulative release of DOX was merely 26.87 ± 2.06% in the absence of GSH even after 72 h. Moreover, both nanoparticles without ditelluride bonds (NP_DOX_ and F-NP_DOX_) showed comparable and tardy drug release pattern regardless of the reductive agent (Figures 4B,C). Together, the boosted drug release demonstrated that the breakage of ditelluride bond of F-TeNP_DOX_ in redox condition, such as GSH, leads to a rapid destruction of nanoparticles and accelerated cargo leakage from the micelle core.

### Cellular Uptake *in vitro*

**Figure 5:**
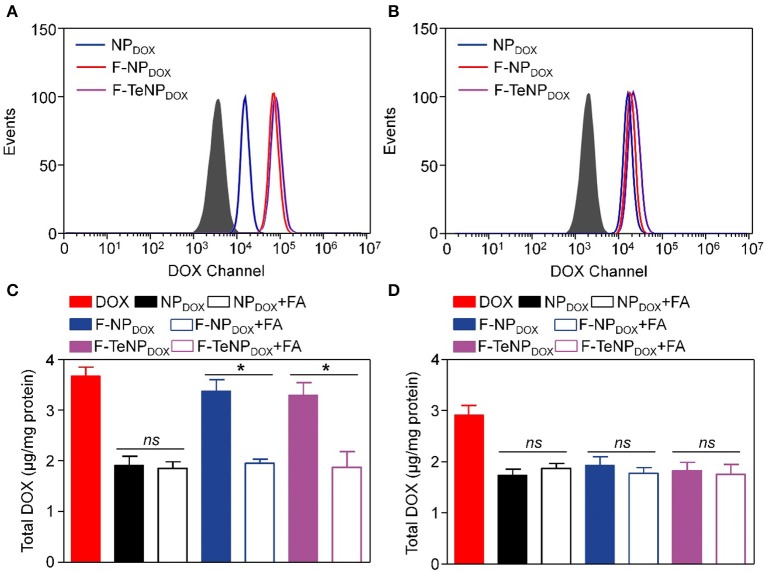
The cellular uptake of NP_DOX_, F-NP_DOX_, or F-TeNP_DOX_ on 4T1 cells **(A)** or NIH3T3 cells **(B)**. Quantitative analyses of DOX content in 4T1 cells **(C)** or NIH 3T3 cells **(D)** after incubation with NP_DOX_, F-NP_DOX_, or F-TeN_PDOX_ for 4 h. [DOX] = 4 μg/mL. *p < 0.05. ns, no significant difference.

For the purpose of evaluating the targeting ability of the nanoparticles modified with folic acid (FA), two types of cell lines were chosen. The 4T1 cell line is over-expressed folate receptors (FR) which can specifically bind to FA while the NIH-3T3 cells do not express the FR were used as the control cell line (Rathinaraj et al., 2015; Han et al., 2016). Furthermore, flow cytometry was performed to compare endocytosis of DOX-loaded nanoparticles (NP_DOX_, F-NP_DOX_, or F-TeNP_DOX_). After incubation with various formulations for 4 h, the intracellular DOX fluorescence was analyzed. As shown in Figure 5A, it is worth noting that the intracellular fluorescence intensity of 4T1 cells treated with F-NP_DOX_ or F-TeNP_DOX_ was much higher than that of NP_DOX_, suggesting the targeting attributes of the FA on the micelle surface. By contrast, no obvious difference can be detected among the three nanoparticles in NIH-3T3 cells, and the mean fluorescence intensity (MFI) value for three nanoparticles was apparently lower than that of 4T1 cells (Figure 5B). Furthermore, the promoted cellular uptake of FA-mediated nanoparticles was further confirmed by the quantitative analysis of internalized DOX content. As expected, the intracellular DOX concentration of NP_DOX_ was 1.91 ± 0.18 μg per mg protein while the F-NP_DOX_ and F-TeNP_DOX_ achieved 3.38 ± 0.23 and 3.29 ± 0.24 μg DOX per mg protein, respectively (Figure 5C). Meanwhile, the drug content was significantly inhibited if adding free FA to the medium because of the competitive effect between the FA-decorated nanoparticles and the free FA. On the other hand, it is worth noting that the intracellular concentration of three nanoparticles showed a negligible difference on NIH-3T3 cells regardless of FA modification (Figure 5D). These results demonstrated that both F-NP_DOX_ and F-TeNP_DOX_ tended to be internalized into 4T1 cells via FA-mediated endocytosis, which was in agreement with previous reports.

**Figure 6:**
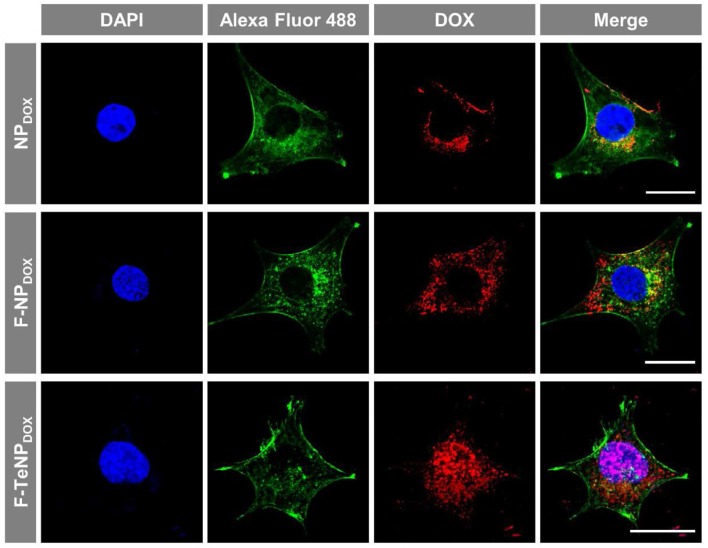
Cellular internalization of NP_DOX_, F-NP_DOX_, or F-TeNP_DOX_ on 4T1 cells. DAPI (blue) and Alexa Fluor 488 phalloidin (green) were used to stain cell nuclei and F-actin, respectively. The scale bar is 20 μm.

Following the FACS and HPLC measurement, the enhanced cellular uptake of targeting nanoparticles was further corroborated using a Zeiss LMS810 confocal laser scanning microscope. The 4T1 cells were incubated with NP_DOX_, F-NP_DOX_, or F-TeNP_DOX_ as described above for 4 h, and the cell nuclei and F-actin were labeled by 4',6-diamidino-2-phenylindole (DAPI) and Alexa Fluor® 488 Phalloidin, respectively. As displayed in Figure 6, a slightly weaker DOX fluorescence was observed in 4T1 cells incubated with non-targeted NP_DOX_. However, a significantly stronger red fluorescence was clearly observed for the cells incubated with F-NP_DOX_ or F-TeNP_DOX_, suggesting the stronger internalization of nanoparticles mediated by FA ligand. Moreover, for cells treated with F-NP_DOX_ or NP_DOX_, the DOX signal were dominantly localized in the cytoplasm. On the contrary, broader red DOX fluorescence was detected in the nucleus of cells which were incubated with F-TeNP_DOX_, indicating the intracellular abundant GSH drive the accelerated DOX release and subsequent transfer to the nucleus. These results confirmed that the ditelluride bond of F-TeNP_DOX_ could be cleaved in intracellular redox microenvironment and therefore the loaded drug can be released effectively.

### Cell Killing Efficiency *in vitro*

**Figure 7:**
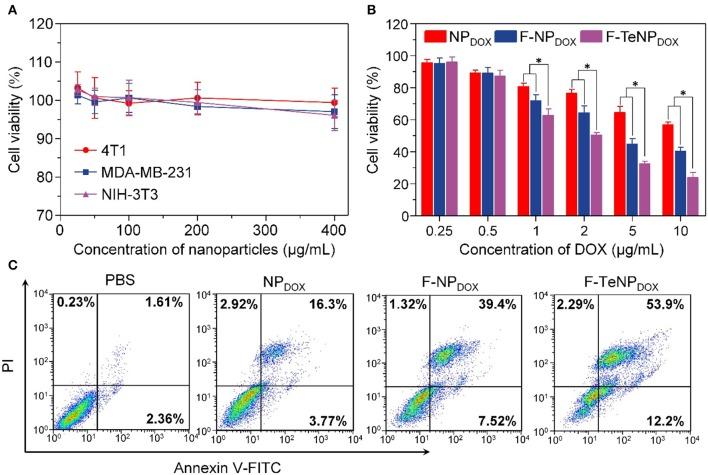
**(A)** MTT assay of F-TeNP on different cell lines (MDA-MB-231, 4T1, and NIH-3T3). The cells were incubated with nanoparticles for 72 h. **(B)** Therapeutic effect of NP_DOX_, F-NP_DOX_, and F-TeNP_DOX_ on 4T1 cells. *p < 0.05. **(C)** 4T1 cell apoptosis induced by various formulations.

Generally, good biocompatibility is the major prerequisite for nanoparticles to be an efficient drug delivery system. Therefore, MDA-MB-231, 4T1 and NIH-3T3 cells were used in a standard MTT assay for 72 h to evaluate the cell viability of blank FA-modified nanoparticles (F-TeNP). As shown in Figure 7A, the cell viabilities showed no significant difference among three cell lines and maintained above 95% even at the highest F-TeNP concentration (400 μg/mL), foreseeing the F-TeNP was biosafe in future application *in vivo*.

Thinking about the cell-killing mechanism of DOX is related to the nucleus entering and its interaction with DNA major groove, the targeted DOX delivery into 4T1 cells and subsequent GSH-triggered drug release would improve the cell growth inhibition and cell apoptosis. Next, the cancer cell-killing efficacy of F-TeNP_DOX_ was studied on 4T1 cells. Following incubation with NP_DOX_, F-NP_DOX_, or F-TeNP_DOX_ for 12 h, cells were incubated with DMEM medium for another 60 h, and the results were shown in Figure 7B. Although the DOX at concentrations of 0.5 μg/mL showed no noticeable cytotoxicity to 4T1 cells, the cell viability gradually decreased when the [DOX] was above 1.0 μg/mL. In comparison with the NP_DOX_, the improved cytotoxicity of F-NP_DOX_ is probably due to the increased DOX content inside the cells via FA-mediated internalization. More importantly, it is worth noting that treatment with F-TeNP_DOX_ decreased the cell viability to 50.35±1.56%, 32.57±1.59% and 23.93±3.14% when the DOX concentration is 2.0, 5.0, and 10.0 μg/mL, respectively.

To further verify the phototoxicity effect of F-TeNP_DOX_, we analyzed cell apoptosis after the treatment using annexin-V-FITC and propidium iodide staining. In comparison with other groups (PBS, NP_DOX_, and F-NP_DOX_), 66.1% of apoptotic cells was observed in the cells treated with F-TeNP_DOX_ (Figure 7C). These results were in agreement with the abovementioned observations, demonstrating that enhanced cell-killing efficiency of F-TeNP_DOX_ is a result of FA-facilitated cellular uptake combined with GSH-responsive cargo release.

### Biodistribution *in vivo*

**Figure 8:**
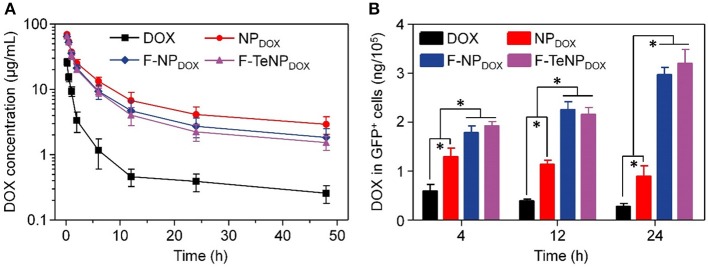
**(A)** Plasma DOX concentration vs. time after *i.v*. injection of free DOX, NP_DOX_, F-NP_DOX_, or F-TeNP_DOX_ (*n* = 4). **(B)** Quantitative analysis of DOX content in GFP-expressing 4T1 cells. *p < 0.05.

With PEGylated shell outside the micelles, NP_DOX_, F-NP_DOX_, or F-TeNP_DOX_ are expected to prolong drug circulation in blood. Accordingly, we then analyzed their pharmacokinetic in female ICR mice. The mice were received a systemic injection of drug-loaded nanoparticles, and free DOX was used as control. The DOX concentration in plasma at 10 min, 0.5, 1, 2, 6, 12, 24, and 48 h was measured by HPLC. In comparison with free DOX, which was cleared rapidly as previous demonstrated, NP_DOX_, F-NP_DOX_, or F-TeNP_DOX_ exhibited a promoted DOX concentration in plasma at each time interval (Figure 8A). After 48 h-post injection, the DOX concentration of NP_DOX_, F-NP_DOX_, or F-TeNP_DOX_ was 11.40–, 7.12–, and 5.95-fold higher than that of free DOX, respectively. Moreover, all of the nanoparticular formulations showed more advanced area under curve (AUC_0−t_) and clearance (CL) (Table S2), suggesting their superior retention in bloodstream.

The prolonged blood circulation of micellar nanoparticles ensured drug molecules greater opportunity to accumulate in tumor tissues via EPR effect. Following the extravasation from tumor blood vessels, FA ligand on F-NP_DOX_ or F-TeNP_DOX_ would facilitate more drug into the tumoral cell (Li et al., 2015). To confirm this hypothesis, we constructed 4T1/GFP xenografts in mice and analyzed intracellular DOX content in GFP-positive tumoral cells. Following the intravenous injection with DOX, NP_DOX_, F-NP_DOX_ or F-TeNP_DOX_, the 4T1/GFP cells were isolated by flow cytometry and intracellular DOX content was quantified using HPLC at different time intervals. As shown in Figure 8B, mice treated with F-NP_DOX_ or F-TeNP_DOX_ displayed ~1.43, 1.95, and 3.44-fold higher DOX retention than that of NP_DOX_ at 4, 12, and 24 h, respectively. Meanwhile, there was no significant difference between the drug content of both targeted nanoparticles, indicating the ditelluride linkage was stable and would not impede micelle biodistribution in the body. These results indicated that the micellar F-TeNP with the PEGylation and FA modification substantially improved drug accumulation in tumoral cells through both passive and active targeting.

### Antitumor Efficiency *in vivo*

**Figure 9:**
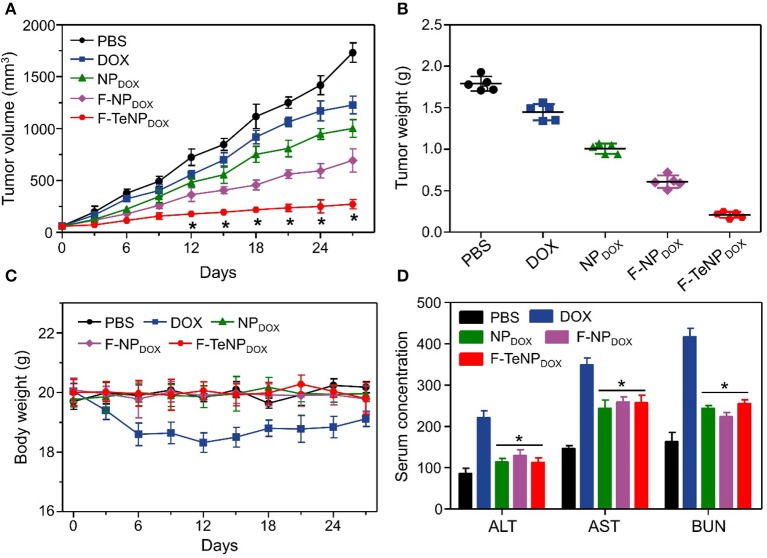
**(A)** Tumor growth curve in 4T1 tumor xenograft-bearing nude mice after various treatments. The injections were performed on days 0, 7, 14, and 21. *p < 0.05. **(B)** The 4T1 tumor weight after the treatment. **(C)** Body weight monitoring of the mice that received treatment with various samples. **(D)** ELISA examination of mouse ALT (U/L), AST (U/L), and BUN (10 μmol/L) in the serum after receiving different treatments. *p < 0.05, vs. DOX.

To investigate *in vivo* tumor therapeutic efficacy, mice bearing 4T1 tumors were used as the model and randomly divided into five groups (five mice each). Each group was received with corresponding intravenous injection with PBS (200 μL), DOX (5.0 mg/kg), NP_DOX_, F-NP_DOX_, or F-TeNP_DOX_ (equivalent DOX dose of 5.0 mg/kg). As shown in Figure 9A, the tumor growth of saline control group was not inhibited, reaching ~1,750 mm^3^ after 27 days. The free DOX and NP_DOX_ showed moderate inhibitory effect. On the contrary, in comparison with F-NP_DOX_ group whereas the ditelluride bond was absence, there was a remarkable growth inhibition in the F-TeNP_DOX_ group, and the tumor volume was only up to 200 mm^3^ at the end of the experiment. After the sacrifice of mice on day 27, the tumor weight analysis further demonstrated that F-TeNP_DOX_ was more effective in tumor growth suppression (Figure 9B). In order to evaluate the biosafety of various treatments, monitoring the body weight of the mice was performed. In Figure 9C, the mice treated with free DOX showed a slight decline of body weight (<10%), and other groups remained relatively stable during the whole observation. In comparison with free DOX, ELISA measurements of ALT, AST, and BUN illustrated that F-TeNP_DOX_ treatment induced less liver and kidney damage (Figure 9D). Moreover, there was no significant difference in blood routine count among PBS, NP_DOX_, F-NP_DOX_, and F-TeNP_DOX_ groups (Table S3), further indicating its low toxicity *in vivo*.

## Conclusions

In this study, we demonstrated the successful preparation of a targeting and redox-responsive delivery system for cancer therapy via FA modified PEG-PCL copolymer with ditelluride linkage. The obtained nanoparticles can efficiently load hydrophobic DOX, and showed rapid drug release triggered by intracellular GSH. Also, F-TeNP_DOX_ could be efficiently internalized into 4T1 cells through FA-mediated endocytosis and achieve sufficient “active-drug” content after redox-responsive micelle dissociation. Moreover, a promoted chemotherapeutic agent accumulation in tumor tissue was observed *in vivo*, leading to a more advanced therapeutic efficiency. Therefore, our work presents a promising drug delivery system based on ditelluride-bridged copolymer, providing a new avenue toward a deeper understanding of precise drug delivery and cancer therapy.

## Data Availability Statement

All datasets generated for this study are included in the article/Supplementary Material.

## Ethics Statement

The animal study was reviewed and approved by Tianjin Medical University Animal Care and Use Committee.

## Author Contributions

CS conceived the ideas, synthesized and characterized delivery system, reviewed, and edited the manuscript. ZP and JZ performed cell and animal experiments and wrote the original draft. All authors discussed the results and commented on the manuscript.

### Conflict of Interest

The authors declare that the research was conducted in the absence of any commercial or financial relationships that could be construed as a potential conflict of interest.
