# Supplementary material for: Ditelluride-Bridged PEG-PCL Copolymer as Folic Acid-Targeted and Redox-Responsive Nanoparticles for Enhanced Cancer Therapy
Source: Front Chem. 2020 Feb 28;8:156. doi: 10.3389/fchem.2020.00156 (PMC7059598; doi:10.3389/fchem.2020.00156)
Supplement: Supplementary file 1 [file Table_1.DOCX]

**Ditelluride-Bridged PEG-PCL Copolymer as Folic Acid-Targeted and Redox-Responsive Nanoparticles for Enhanced Cancer Therapy**

Zekun Pang^1^ , Jiayan Zhou^1^ and Chunyang Sun^1,^*

^1^ Department of Radiology and Tianjin Key Laboratory of Functional Imaging, Tianjin Medical University General Hospital, Tianjin 300052, P.R. China

**Methods**

Nuclear magnetic resonance (NMR) analyses were measured using a Bruker AV300 spectrometer. The size and size distribution of the nanoparticles were analyzed using a Malvern Zetasizer Nano ZS90 apparatus with a He–Ne laser (633 nm) and 90° collecting optics.

**Cell Lines and tumor models**

The 4T1 mouse breast tumor cells and NIH-3T3 cells from the American Type Culture Collection (ATCC) were cultured in Dulbecco's Modified Eagle Medium (DMEM, Gibco, Grand Island) supplemented with 10% fetal bovine serum (FBS, Hyclone, Thermo Scientific, USA). All cells were incubated at 37 °C in a 5% CO_2_ atmosphere. Female BALB/c mice (4 weeks old) were purchased from the Vital River Laboratory Animal Technology Co., Ltd. (Beijing, China). All *in vivo* experiments were performed according to Tianjin Medical University Guidelines for Animal Research, and were approved by Tianjin Medical University Animal Care and Use Committee. To establish a 4T1/GFP xenograft tumor model, 4T1/GFP cells (1 × 10^6^ for each mouse) were injected into the mammary fat pat of female BALB/c mice.


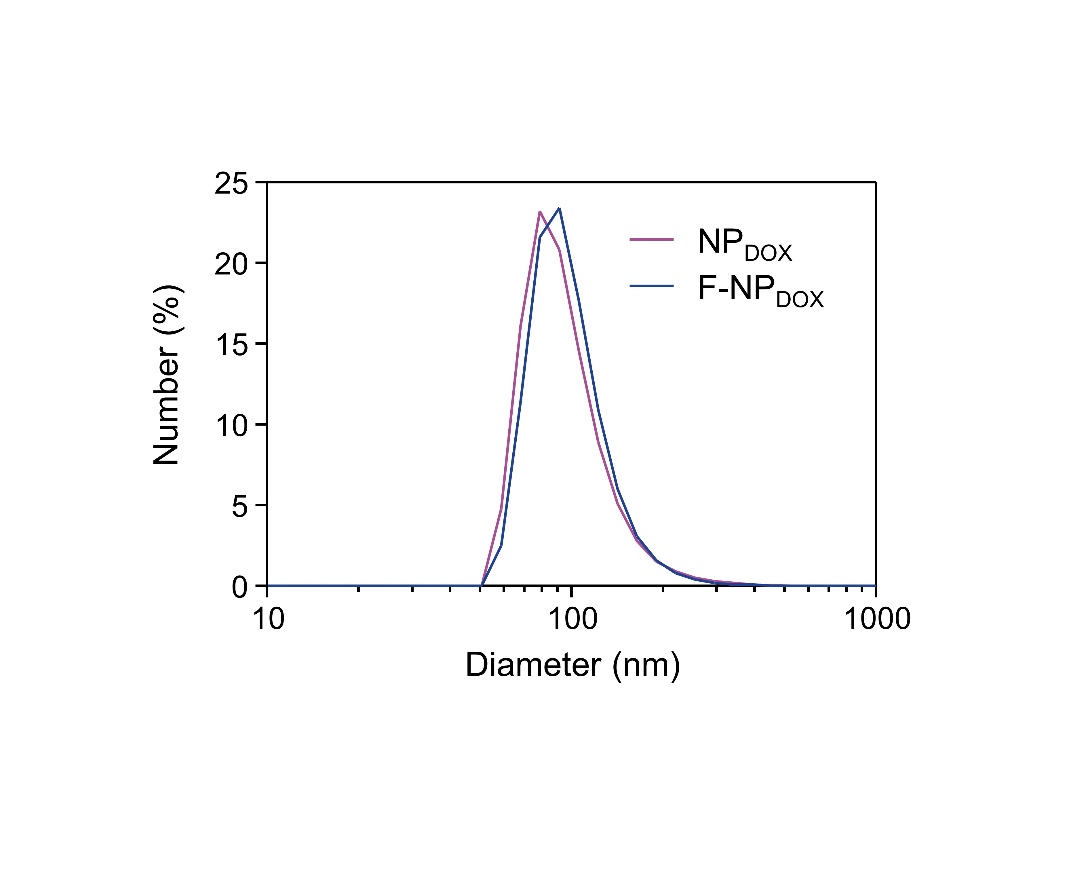


**Figure S1.** Size distribution of NP_DOX_ and F-NP_DOX_.

|  | Mean size (nm) | Zeta potential (mV) | PDI | DLC (%) | EE (%) |
| --- | --- | --- | --- | --- | --- |
| NP_DOX_ | 97.9 | -11.9 | 0.166 | 5.45 | 57.7 |
| F-NP_DOX_ | 101.0 | -13.2 | 0.182 | 5.13 | 54.1 |
| F-TeNP_DOX_ | 96.5 | -10.7 | 0.158 | 5.74 | 60.9 |

**Table S1.** Characterization of NP_DOX_, F-NP_DOX_, F-TeNP_DOX_. The mean size, zeta potential and PDI were determined by DLS.

|  | AUC_0-t_ (μg/mL*h) | CL (mL/h) |
| --- | --- | --- |
| DOX | 60.202±25.792 | 2.388±0.348 |
| NP_DOX_ | 355.900±17.802 | 0.494±0.061 |
| F-NP_DOX_ | 267.958±31.588 | 0.515±0.178 |
| F-TeNP_DOX_ | 242.037±13.426 | 0.624±0.180 |

**Table S2.** Pharmacokinetic parameters of these formulations after intravenous administration.

|  | PBS | NP_DOX_ | F-NP_DOX_ | F-TeNP_DOX_ |
| --- | --- | --- | --- | --- |
| WBC | 4.69±0.22 | 4.45±0.31 | 4.39±0.29 | 4.90±0.56 |
| RBC | 10.22±0.72 | 10.02±0.53 | 9.78±0.87 | 10.65±0.36 |
| HGB | 161.25±3.98 | 166.13±4.80 | 157.50±5.55 | 161.86±4.05 |
| HCT | 35.03±2.42 | 34.00±2.73 | 34.96±2.87 | 35.71±3.09 |
| MCV | 33.93±2.59 | 32.89±1.17 | 35.31±2.63 | 33.08±2.98 |
| MCH | 16.71±0.44 | 16.65±0.57 | 16.44±0.37 | 16.8±0.22 |

**Table S3.** Routine blood analysis of BALB/c mice treated with different formulations. WBC: white blood cells, RBC: red blood cells, HGB: hemoglobin, HCT: hematocrit, MCV: mean corpuscular volume, MCH: mean corpuscular hemoglobin.
